# Effects of a WeChat Mini-Program on Human Milk Feeding Rates in a Neonatal Intensive Care Unit During the COVID-19 Pandemic

**DOI:** 10.3389/fped.2022.888683

**Published:** 2022-06-21

**Authors:** Chengyao Jiang, Xue Chu, Zhangbin Yu, Xiaohui Chen, Jun Zhang, Shuping Han

**Affiliations:** Department of Paediatrics, Women’s Hospital of Nanjing Medical University, Nanjing Maternity and Child Health Care Hospital, Nanjing, China

**Keywords:** human milk expression, human milk feeding, WeChat mini-program, lactation education, neonatal intensive care, COVID-19

## Abstract

**Objective:**

We investigated changes in maternal daily milk pumping frequency and milk volume per expression and their derived lactation indicators, as well as human milk (HM) feeding status with a focus on amount and rates in preterm infants admitted to the neonatal intensive care unit (NICU) after using a WeChat mini-program during the 2019 coronavirus (COVID-19) pandemic.

**Methods:**

The study was conducted with 482 mothers and their 544 babies. We prospectively enrolled mothers and infants with birth weight <1,500 g or gestational age <32 weeks born in 2020, and retrospectively included the same population in 2019. All study subjects were classified into three subgroups: pre-pandemic (PP, 2019), early pandemic (EP, January to April 2020), and late pandemic (LP, May to December 2020). From 1 January 2020, mothers recorded in an online pumping diary using the WeChat mini-program. We obtained the infants’ feeding information from an online database for analysis.

**Results:**

Maternal lactation indicators did not change significantly. However, 56.7% (139/245) of mothers achieved milk volume ≥500 ml/day (CTV) in PP, 58.9% (33/156) in EP, and a slight increase to 60.7% (91/150) in LP. Maternal pumping frequency remained about eight times/day. In LP, daily milk volume was higher than the other two periods from day 4, and mothers achieved CTV by day 12, which was achieved in the other two groups by 13–14 days. There were several statistical differences in the amount and rates of feeding between the groups, particularly about HM and donor milk feeding, with the vast majority being decreased during EP, while during LP they returned to PP levels. Pleasingly, the median average daily dose of HM at 1–28 days was highest in LP (LP, 87.8 vs. PP, 75.5 or EP, 52.6 ml/kg/day, *P*_corrected_ < 0.001). In addition, most categorical feeding indicators decreased in EP and recovered in LP.

**Conclusion:**

An education model based on the WeChat program could aid lactation education and management in mothers of preterm infants to maintain healthy lactation. The model, together with optimized management strategies, can ensure that the HM feeding rate is not compromised in vulnerable high-risk infants during NICU hospitalization in a public health emergency, like the COVID-19 pandemic.

## Introduction

Human milk (HM) feeding, especially consuming the mother’s own milk (MOM), is recognized as the gold standard for newborns (1). MOM can provide newborns with energy, the nutrients required for growth, essential microbes, and other beneficial bioactive components, such as HM oligosaccharides and immunoglobulins (2). It can also reduce short-term complications, such as neonatal necrotizing enterocolitis (NEC), chronic lung diseases, retinopathy of prematurity, late-onset sepsis, and more significantly, it promotes neurodevelopment in the long term (3–5). Donor milk (DM) is recommended by many international agencies, such as the World Health Organization, to feed vulnerable infants as the suboptimal option when MOM is not available (6). Even so, DM is far superior to formula, particularly in reducing NEC (7). The World Health Organization recommends that neonates should be breastfed exclusively for at least 6 months and then continue, even with complementary food, for 2 years or more (8).

Successful HM feeding in the NICU must be supported by an adequate milk supply from the mother. However, mothers of preterm infants tend to face more difficulties in the initiation and maintenance of lactation than mothers of term infants. The most important and prevalent problem for these mothers is long-term mother–infant separation (9). When direct feeding is interrupted, the natural stimulus of neonatal sucking is lost, and mothers can then only express milk by breast pumps or by hand (10, 11). Many mothers have maternal complications that affect normal lactation, such as preeclampsia and obesity. In addition, treatment with medications such as antenatal corticosteroids also aggravates the delay of lactation (12).

At the end of the year 2019, COVID-19 broke out in Wuhan, China, and subsequently spread widely overseas as a global pandemic (13). This undoubtedly further hindered the promotion of breastfeeding in the NICU. Increased social distancing reduced mothers’ lactation support from family and society, suspending parents’ NICU visits reduced skin-to-skin contact that promotes maternal lactation and preterm infant physiological health (14, 15), and traffic restrictions caused considerable difficulties in delivering milk and donating milk to the NICU. Together, these adverse conditions have aggravated mothers’ anxiety and can even lead to depression (16), both of which interfere with normal lactation. At the same time, traditional bedside breastfeeding education has also been greatly affected.

The emergence of mobile technology has opened new ideas for health management (17), and is used to manage high blood pressure, diabetes, smoking cessation, and promotion of breastfeeding (18–21). In the context of maintaining social distancing due to COVID-19, mobile education is undoubtedly the best breastfeeding intervention tool to break social distancing. Using WeChat, a mobile phone app with 1.15 billion monthly active users as of 2019 (3, 11), we developed a mini-program named “Ning BX breastfeeding” to promote breastfeeding in the NICU (22).

It is not possible to know the specific time when breastfeeding was affected, nor it is possible to conclude when the breastfeeding environment completely returned to normal. Therefore, we synchronized the development timeline of the Wuhan epidemic with local management policies to define three stages: pre-pandemic (PP, 2019), early pandemic (EP, January to April 2020), and late pandemic (LP, May to December 2020). The purpose of this study is to compare maternal HM expression behavior in the above three stages and, more importantly, the HM feeding amount and rates of the preterm infants admitted to the NICU using quantitative and categorical evaluation indicators.

## Materials and Methods

### Study Cohort and Ethics

This study prospectively enrolled mothers and their infants born in 2020 and retrospectively included the same population in 2019 as well. It was carried out in the NICU of Women’s Hospital of Nanjing Medical University, a tertiary baby-friendly hospital in Jiangsu Province, China. Inclusion criteria were: (a) infants’ birth weight <1,500 g while gestational age <35 weeks or gestational age <32 weeks while birth weight <2,000 g; (b) newborns admitted to the NICU within 24 h after birth; (c) mother ≥ 18 years old; and (d) mothers were willing to participate in the study and signed the informed consent form. Exclusion criteria were: (a) premature infants discharged, transferred, abandoned, or who died within 14 days; (b) preterm infants who failed to achieve full enteral feeding during hospitalization; (c) incomplete data; or (d) newborns or mothers with breastfeeding contraindications.

**FIGURE 1:**
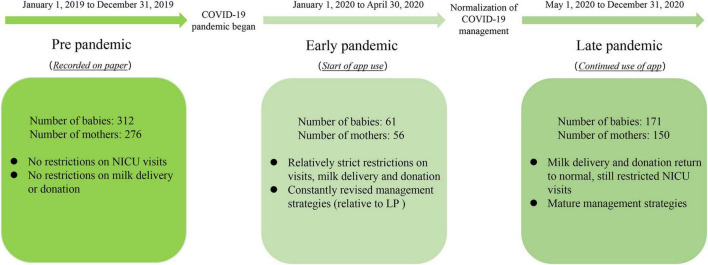
Study timeline related to the COVID-19 pandemic. The boxes contain the number of preterm infants and their mothers enrolled, as well as specific NICU management strategies. NICU, neonatal intensive care unit; LP, late pandemic.

We based our timeline on the Wuhan epidemic and local management policies. Accordingly, 482 mothers and their 544 babies were classified into three subgroups: PP (2019, no mini-program used), EP (January to April 2020, mini-program used), or LP (May to December 2020, mini-program used). The specific timeline is shown in Figure 1. The Ethics Committee of the Women’s Hospital of Nanjing Medical University approved this protocol (Reference No. [2020] KY-017).

### Lactation Education System

#### Antepartum

The “Fetal University” course established by our hospital is mainly aimed at pregnant mothers. Parents are provided knowledge about breastfeeding offline at 3 pm every Friday, which can be checked on our hospital’s WeChat Official Accounts.

#### Postpartum in Obstetrics

When mothers return to the obstetric ward postpartum, they rent or bring their own electric breast pump and first express breast milk under the guidance of a trained obstetric nurse. They are also taught breast milk expression skills and appropriate daily pumping frequency. Daily pumping times and milk volume are recorded by the nurse on a bedside record sheet.

### Postpartum in the NICU

#### Education Time

Mothers who delivered during the day are usually educated that day, and those who delivered in the evening are taught the following day (target within 8 h of delivery).

#### Education Location

(a) Before the pandemic, we went to the bedside in the obstetric ward to teach mothers and (b) during the pandemic, we arranged a separate education room to prevent and control COVID-19.

#### The NICU Lactation Education Team

Includes at least one trained neonatologist and neonatal nurse.

#### Education Content:

(a) Emphasize that HM feeding has substantial implications for preterm infants; (b) describe the long-term benefits of maintaining lactation; (c) require mothers to express breast milk at least 6–8 times daily with two milking intervals of no more than 5 h; (d) correct expression of breast milk and equipment disinfection procedures; (e) use teaching models to demonstrate the breast pump and accurate pumping skills; (f) collection, transportation, and storage of breast milk; And (g) inform mothers about the newborn’s general condition (mainly milk consumption and body weight).

#### Education Procedure:

Before the pandemic, mothers recorded their daily milk pumping frequency and volume data on paper (at least 14 days), and sent it to us in the form of text via WeChat Official Accounts after we convey the educational content at the mothers’ bedside. To prevent and control COVID-19, we changed the education procedure. We generally ask the father to bring the mother’s mobile phone (with consent), and then scan the code to log in to their personal account. The educator uploads the baby’s first photo and text of lactation information in the Growth Record on the medical staff side. The first operation process is demonstrated by the educator. Then, the father is required to complete other previous lactation records in the obstetric ward when he has learned the whole process and we particularly emphasize noting when mothers first experience breast fullness, swelling, and milk leakage. As mothers know the lactation details best and have more time to record, we ask fathers to teach mothers the operation process and parents can participate in the recording activities together. Educators will put the operation video on our WeChat Official Accounts and inform parents that we can be contacted by the Growth Record on the mini-program or by telephone if they have any problems. Educators will check the mini-program daily and contact mothers with daily pumping times <6 or a slow milk volume increase through the mini-program or by phone to understand the problem and help solve it. We created an additional incentive mechanism for mothers who actively participated in data recording and sent 2–4 photographs of their babies every 2–3 days with encouraging words. On day 14, we thank the mother for her efforts for her children and encourage them to maintain adequate milk production through the Growth Record. More importantly, they can still contact us through the mini-program or by phone, and we may recommend mothers to the Lactation Clinic if necessary. For more details on the features of the WeChat mini-program, please refer to the previously published study protocol of our group (22).

### Measurement

The following neonatal baseline variables were measured: birth weight (BW), gestational age (GA), gender, singleton or multiple births, length of hospital stay, hospitalization costs, 1-min Apgar score, feeding intolerance, neonatal necrotizing enterocolitis (≥stage 2 or surgically treated), late-onset sepsis (clinical signs of sepsis with positive blood culture >3 days from birth), retinopathy of prematurity (≥stage 3 or surgically treated), or bronchopulmonary dysplasia (moderate to severe).

The maternal baseline variables were as follows: maternal age, spouse age, advanced maternal age (childbearing age ≥35 years), delivery methods (vaginal delivery or cesarean section), parity, and hypertensive disorders of pregnancy.

Mothers’ daily milk pumping frequency and milk volume per expression (1–14 days), and their derived variables (i.e., maternal lactation indicators) were recorded: first milk expression time (first milking time by pump or hand, h); first lactation time (first milk expression ≥ 1 ml, h); OOL II-F (onset of lactogenesis II in feeling, h) refers to the first time mothers feel breast fullness, swelling, and milk leakage after delivery; OOL II-Q (onset of lactogenesis II in quantitative, h) refers to the first time point when two consecutive milk volumes are ≥20 ml; and achieve CTV means the mothers achieve daily milk yield ≥ 500 ml within 14 days.

During hospitalization, daily feeding for preterm infants includes HM (consisting of MOM and DM) and formula milk (FM). The following variables were derived: average MOM-DD (average daily dose of MOM), average DM-DD (average daily dose of DM), average HM-DD (average daily dose of HM), cumulative MOM-PCT ({sum of MOM/[sum of HM + sum of FM]} × 100%), cumulative DM-PCT ({sum of DM/[sum of HM + sum of FM]} × 100%), and cumulative HM-PCT ({sum of HM/[sum of HM + sum of FM]} × 100%). For more details about these six indicators, refer to the study of Bigger et al. (23). HM-Ever and MOM-Ever indicate that infants ever received HM or MOM. Exclusive HM-DC (or MOM-DC) means those who were still receiving HM (or MOM) at NICU discharge. Other categorical variables are presented in Table 5. Finally, we also recorded the time to achieve the first full enteral feeding (≥120 ml/kg/day).

All calculations of the above-mentioned variables involving time are based on neonatal birth time.

### Data Collection

The following data were entered into an online database named ‘‘NingBX neonatal perinatal network’’^1^ by well-trained researchers: baseline variables for neonates and mothers, feeding status of preterm infants from 1 to 28 days and at discharge, and newborns’ daily weight within 28 days. Data on mothers’ daily milk pumping frequency and milk volume per expression from 1 to 14 days were collected through the WeChat mini-program and automatically linked to the online platform mentioned earlier. We then exported data in the background for research analysis. Data were strictly managed to ensure privacy.

### Statistical Analysis

Continuous variables were described as M (P_25_, P_75_). Differences between two groups were compared using the Mann–Whitney U-test and between three groups using the Kruskal–Wallis test. Categorical variables were described as frequency and percentage, and comparisons between groups were performed using a contingency tables-based chi-square test or Fisher’s exact test. Bonferroni correction was used for pairwise comparisons between multiple groups. We used IBM SPSS Statistics 26.0.0 for data analysis. *P*-value < 0.05 was considered significant (two-tailed).

## Results

The respective number of infants in each group was 312, 61, and 171, and the respective number of mothers was 276, 56, and 150. Some data in PP were collected retrospectively, and thus the number of mothers was reduced to 245 in 2019 (245/276) for the comparison of maternal lactation behavior due to incomplete data. Basic characteristics, comorbidities of the enrolled infants, and maternal demographic data are presented in Table 1.

**TABLE 1:** Basic characteristics and comorbidities of the enrolled infants and maternal demographic data.

Infants	PP (n = 312)	EP (n = 61)	LP (n = 171)	H/χ^2^	*P*-value
Gestational age (weeks)	30.0 (28.0, 31.0)	30.0 (28.0, 31.0)	30.0 (28.0, 31.0)	0.828	0.661
Birth weight (g)	1,305.0 (1,142.5, 1,540.0)	1,310.0 (1,055.0, 1,520.0)	1,350.0 (1,130.0, 1,540.0)	0.949	0.622
Sex (male)	157 (50.3)	35 (57.4)	97 (56.7)	2.318	0.314
Multiple birth	91 (29.2)	13 (21.3)	52 (30.4)	1.905	0.386
Length of hospital stay (days)	36.0 (28.0, 46.8)	42.0 (30.0, 56.0)	36.0 (28.0, 49.0)	4.148	0.126
Hospitalization costs (yuan)	71,250.5 (51073.7, 99783.0)	79,977.3 (54,065.1, 139,944.6)	72,873.9 (51,020.3, 107,441.3)	4.765	0.092
1-min Apgar score	9.0 (8.0, 10.0)	10.0 (8.0, 10.0)	9.0 (8.0, 10.0)	0.056	0.972
Feeding intolerance	113 (36.2)	28 (45.9)	53 (31.0)	4.454	0.108
Necrotizing enterocolitis	27 (8.7)^a^	2 (3.3)	4 (2.3)^b^	8.667	0.013
Late-onset sepsis	11 (3.5)	3 (4.9)	4 (2.3)	1.034	0.596
Severe ROP or ROP surgery	5 (1.6)	3 (4.9)	7 (4.1)	3.681	0.159

**Mothers**	**PP (n = 276)**	**EP (n = 56)**	**LP (n = 150)**	**H/χ^2^**	***P*-value**

Maternal age (years)	31.0 (28.0, 34.0)	30.0 (27.0, 31.0)	31.0 (28.0, 33.0)	3.066	0.216
Spousal age (years)	32.0 (29.0, 35.0)	31.0 (29.0, 35.0)	32.0 (29.0, 35.0)	1.080	0.583
Advanced maternal age	65 (23.6)	8 (14.3)	26 (17.3)	3.820	0.148
Cesarean section	164 (59.4)	31 (55.4)	84 (56.0)	0.632	0.729
Primipara	189 (68.5)	36 (64.3)	103 (68.7)	0.414	0.813
HDP	38 (13.8)	12 (21.4)	20 (13.3)	2.449	0.294

*HDP, hypertensive disorders of pregnancy; ROP, retinopathy of prematurity. Data are presented as n (%) or M (P25, P75).*

*P-values are results from Kruskal–Wallis or Chi-square tests of all groups.*

*Different superscript letters between the two groups indicate a difference after Bonferroni correction.*

**TABLE 2:** Maternal lactation indicators.

Variables	PP (n = 245)	EP (n = 56)	LP (n = 150)	χ^2^/Z	P-value
Achieved CTV	139 (56.7)	33 (58.9)	91 (60.7)	0.740	0.602
First milk expression time (h)	–	7.0 (4.3, 10.0)	8.0 (5.0, 13.0)	−0.481	0.631
First lactation time (h)	–	33.0 (17.0, 61.8)	44.5 (24.0, 60.5)	−0.930	0.352
OOL II-F (h)	–	70.5 (51.5, 99.5)	63.5 (50.0, 85.8)	−1.024	0.306
OOL II-Q (h)	–	70.0 (52.3, 89.3)	68.0 (56.0, 82.8)	−0.328	0.743

*A total of four mothers in EP and six in LP did not show signs of OOL II-F (breast fullness, swelling, and milk leakage).*

*A total of two mothers in LP failed to achieve OOL II-Q.*

*Data are presented as n (%) or M (P25, P75).*

**TABLE 3:** Daily milk pumping frequency and milk volume on day 1–14 postpartum.

Postpartum days	Pumping frequency				Milk volume			
		
	PP (n = 245)	EP (n = 56)	LP (n = 150)	*P*-value	PP (n = 245)	EP (n = 56)	LP (n = 150)	P-value
1	3 (2, 5)^b^	1 (0, 3)^a^	1 (0, 2)^a^	<0.001	0.0 (0.0, 0.3)	0.0 (0.0, 0.0)	0.0 (0.0, 0.1)	0.109
2	7 (6, 8)^b^	6 (4, 7)^a^	7 (3, 8)	0.002	0.5 (0.0, 5.0)	0.0 (0.0, 5.0)	0.5 (0.0, 6.7)	0.573
3	8 (7, 8)	7 (6, 8)	8 (7, 8)	0.066	15.0 (3.0, 60.0)	16.0 (0.0, 73.8)	14.0 (1.2, 58.5)	0.794
4	8 (7, 8)^b^	8 (7, 9)	8 (7, 8)^a^	0.027	110.0 (40.0, 240.0)	122.0 (29.8, 278.5)	144.5 (43.0, 270.0)	0.425
5	8 (7, 8)^b^	8 (7, 8)	8 (7, 8)^a^	0.011	210.0 (100.0, 320.0)	183.0 (90.8, 402.5)	245.0 (139.0, 391.3)	0.151
6	8 (7, 8)	8 (7, 8)	8 (7, 8)	0.161	260.0 (160.0, 420.0)	235.5 (130.3, 480.0)	315.0 (183.3, 506.3)	0.126
7	8 (7, 8)	8 (7, 8)	8 (7, 8)	0.423	330.0 (200.0, 480.0)	298.5 (178.5, 553.3)	362.0 (198.4, 586.3)	0.298
8	8 (7, 8)	8 (7, 8)	8 (7, 8)	0.474	360.0 (240.0, 560.0)	334.0 (242.8, 555.0)	407.5 (237.8, 641.3)	0.167
9	8 (7, 8)	8 (7, 8)	8 (7, 8)	0.697	390.0 (250.0, 560.0)	405.0 (251.3, 708.8)	465.0 (268.0, 731.3)	0.100
10	8 (7, 8)	8 (7, 9)	8 (7, 8)	0.783	420.0 (295.0, 640.0)	447.5 (305.0, 681.3)	480.0 (290.0, 741.3)	0.332
11	8 (7, 8)	8 (7, 8)	8 (7, 8)	0.598	450.0 (300.0, 645.0)	445.0 (320.3, 761.3)	489.5 (293.8, 791.3)	0.436
12	8 (7, 8)	8 (7, 8)	8 (7, 8)	0.761	460.0 (325.0, 700.0)	488.0 (335.0, 706.3)	560.0 (299.5, 752.5)	0.465
13	8 (7, 8)	8 (7, 8)	8 (7, 8)	0.896	490.0 (325.0, 700.0)	522.5 (313.3, 795.0)	557.5 (328.8, 780.0)	0.658
14	8 (7, 8)	8 (7, 8)	8 (7, 8)	0.396	500.0 (350.0, 720.0)	517.5 (293.8, 837.5)	545.0 (342.5, 781.3)	0.848

*Pairwise comparisons were Bonferroni corrected, and two groups with different letters are statistically different.*

*Data are presented as M (P25, P75).*

**TABLE 4:** Comparison of quantitative feeding indicators for preterm infants in NICU hospitalization: amount and rates.

	PP (n = 312)	EP (n = 61)	LP (n = 171)	H	*P*-value
**D1-7**					
Average MOM-DD (ml/kg/day)	8.7 (2.9, 16.0)	6.8 (1.8, 17.4)	7.1 (1.9, 15.6)	1.045	<0.593
Average DM-DD (ml/kg/day)	4.3 (2.5, 9.2)^b^	1.8 (0.0, 6.8)^a^	6.0 (2.0, 14.5)^b^	21.174	<0.001
Average HM-DD (ml/kg/day)	16.9 (9.5, 25.1)	15.8 (4.6, 24.5)	18.0 (8.9, 27.5)	3.088	0.214
Cumulative MOM-PCT	66.4 (27.0, 83.3)	72.0 (21.2, 87.1)	49.4 (16.7, 82.4)	3.641	0.162
Cumulative DM-PCT	32.5 (16.4, 70.3)^b^	15.4 (0.0, 50.4)^a^	44.5 (17.2, 82.5)^b^	20.765	<0.001
Cumulative HM-PCT	100.0 (100.0, 100.0)^b^	100.0 (89.9, 100.0)^a^	100.0 (100.0, 100.0)^b^	53.589	<0.001
**D1-14**					
Average MOM-DD (ml/kg/day)	32.3 (16.4, 49.3)	29.2 (5.9, 48.3)	31.7 (14.3, 49.8)	1.387	0.500
Average DM-DD (ml/kg/day)	3.6 (1.6, 14.6)^b^	1.7 (0.2, 9.6)^a^	6.1 (1.8, 22.4)^b^	15.778	<0.001
Average HM-DD (ml/kg/day)	45.4 (29.0, 60.0)	39.5 (19.4, 59.6)^a^	49.5 (27.2, 67.5)^b^	6.898	0.032
Cumulative MOM-PCT	87.3 (62.2, 96.0)	89.9 (38.5, 97.4)	82.2 (47.0, 94.8)	4.567	0.102
Cumulative DM-PCT	12.4 (3.9, 33.5)^b^	6.0 (0.6, 32.9)^a^	17.3 (4.9, 51.9)^b^	12.574	0.002
Cumulative HM-PCT	100.0 (100.0, 100.0)^b^	100.0 (95.7, 100.0)^a^	100.0 (100.0, 100.0)^b^	52.950	<0.001

**D1-28**	**PP (n = 235)**	**EP (n = 49)**	**LP (n = 131)**	**H**	***P*-value**

Average MOM-DD (ml/kg/day)	61.8 (40.0, 87.6)	43.1 (27.1, 80.0)^b^	69.2 (44.3, 91.8)^a^	8.712	0.013
Average DM-DD (ml/kg/day)	3.5 (1.0, 12.0)	1.8 (0.2, 15.3)^b^	5.6 (1.1, 19.8)^a^	8.479	0.014
Average HM-DD (ml/kg/day)	75.5 (47.8, 100.8)^a^	52.6 (36.7, 87.5)^b^	87.8 (60.2, 103.7)^c^	17.491	<0.001
Cumulative MOM-PCT	93.2 (76.8, 98.6)	75.7 (38.4, 98.2)	89.8 (67.4, 98.0)	5.209	0.074
Cumulative DM-PCT	4.8 (1.2, 16.2)	4.1 (0.3, 17.0)	6.5 (1.6, 22.3)	5.493	0.064
Cumulative HM-PCT	100.0 (100.0, 100.0)^b^	98.9 (63.2, 100.0)^a^	100.0 (100.0, 100.0)^b^	35.647	<0.001

*Pairwise comparisons were Bonferroni corrected, and two groups with different letters are statistically different. Data are presented as M (P25, P75).*

**TABLE 5:** Comparison of categorical feeding indicators for preterm infants in NICU hospitalization.

Variables	PP (n = 312)	EP (n = 61)	LP (n = 171)	Fisher/χ^2^/H	*P*-value
MOM-Ever within 1 day	2 (7.1)	2 (28.6)	1 (5.6)	–	0.199
HM-Ever within 1 day	26 (92.9)	5 (71.4)	16 (88.9)	–	0.257
MOM-Ever within 3 days	125 (42.8)	24 (42.1)	72 (44.2)	0.109	0.947
HM-Ever within 3 days	292 (100.0)^a^	47 (82.5)^b^	161 (98.8)^a^	39.279	<0.001
MOM-Ever within 7 days	267 (86.7)	50 (83.3)	143 (83.6)	1.043	0.594
HM-Ever within 7 days	308 (100)^a^	57 (95)^b^	170 (99.4)	–	0.002
Cumulative HM-PCT ≥ 50% within 14 days	310 (99.4)^a^	56 (91.8)^b^	171 (100.0)^a^	–	<0.001
Cumulative MOM-PCT ≥ 50% within 14 days	253 (81.1)	44 (72.1)	124 (72.5)	5.728	0.057
HM-DD ≥ 50ml/kg/d within 28 days*	170 (72.3)^a^	26 (53.1)^b^	107 (81.7)^a^	14.946	0.001
Exclusive MOM feeding within 28 days*	4 (1.7)	3 (6.1)	2 (1.5)	–	0.145
Exclusive HM feeding within 28 days*	192 (81.7)^a^	21 (42.9)^b^	108 (82.4)^a^	37.755	<0.001
Exclusive MOM-DC	260 (83.3)	47 (77.0)	146 (85.4)	2.242	0.326
Exclusive HM-DC	280 (89.7)^a^	48 (78.7)^b^	152 (88.9)	6.110	0.047
Time to achieve full enteral feeding (days)	15 (12, 21)	15 (12, 22)	14 (11, 19)	6.607	0.037

*Pairwise comparisons were Bonferroni corrected, and two groups with different letters are statistically different. Data are presented as n (%) or M (P25, P75).*

*Infants with a hospital stay <28 days were excluded, as in Table 4.

The incidence of NEC was lower in the LP group than in the PP group (2.3 vs. 8.7%, *P*_corrected_ < 0.05). However, the majority of NEC cases occurred after postnatal day 14 (18/33) and some after 28 days (7/33), so the effect of NEC on neonatal feeding should not cause obvious differences between groups during our study period (both *P*_corrected_ > 0.05). No other variables of infants and mothers showed statistical differences. But in EP, the length of hospital stay was longer [EP, 42.0 (30.0, 56.0) vs. PP, 36.0 (28.0, 46.8) or LP, 36.0 (28.0, 49.0) days, P = 0.126] and hospitalization costs were higher [EP, 79,977.3 (54,065.1, 139,944.6) vs. PP, 71,250.5 (51,073.7, 99,783.0) or LP, 72,873.9 (51,020.3, 107,441.3) yuan, *P* = 0.092].

Data on maternal lactation indicators are presented in Table 2. Five indicators were not significantly different between the three groups or between EP and LP. A total of 56.7% (139/245) of mothers achieved CTV in PP, 58.9% (33/56) in EP, and 60.7% (91/150) in LP. The median first milk expression time was 7.0 h for EP and 8.0 h for LP. Mothers in EP took 33.0 h to produce a milk volume >1 ml for the first time, and this was 44.5 h in LP. Mothers in EP took a median of 70.5 h to achieve OOL II-F, while this was 63.5 h in LP. The time taken to achieve OOL II-Q was 70.0 h and 68.0 h in EP and LP, respectively. It is important to note that 10 mothers did not show signs of OOL II-F within 14 days (four in EP and six in LP) and also two mothers failed to achieve OOL II-Q (both in LP).

**FIGURE 2:**
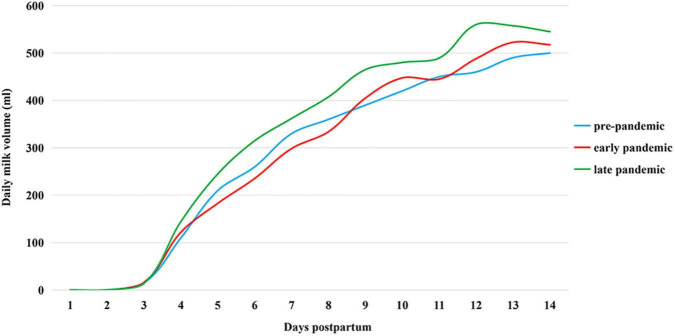
Maternal milk volume in the two weeks postpartum in 2019–2020.

Mothers’ daily milk expression frequency and milk volume during the 2 weeks after delivery are presented in Table 3 and Figure 2. On postpartum days 1, 2, 4, and 5, there was a significant difference between groups in the number of daily milk pumping times (*P* < 0.001, *P* = 0.002, *P* = 0.027, and *P* = 0.011, respectively), with the first 2 days being less frequent in EP and LP. However, by days 4–5, the frequency was higher in the LP than in the PP period. The median daily pumping frequency of mothers remained at eight for the rest of the study period (all *P* > 0.05). The mothers’ daily milk volume showed an increasing trend with time. From the 4th day, milk volume in LP was higher than in the other two groups. However, the change in milk yield among the three groups was not statistically significant. The other findings based on median daily pumped milk volume were as follows: from day 2 to day 3, all three groups of mothers began to show a significant trend in increasing milk volume, from near 0 to 14-16 ml; EP and LP groups showed the largest volume increase every other day on days 3–4 (106 and 130.5 ml, respectively), while PP showed the largest increase of 100 ml on days 4–5. On day 14, the median volume was more than 500 ml in the three groups, while on the 12th day of LP, it exceeded this value.

This study majorly investigated changes in the amount and rates of breastfeeding in the three groups, according to premature infants’ length of hospital stay (1–7 days, 1–14 days, and 1–28 days), as presented in Table 4. From days 1–7, there were differences in average DM-DD, cumulative DM-PCT, and cumulative HM-PCT between the groups (all *P* < 0.001). These were all lowest in EP [1.8 (0.0, 6.8), 15.4 (0.0, 50.4), and 100.0 (89.9, 100.0) ml/kg/day, respectively, all *P*_corrected_ < 0.001)]. From days 1 to 14, these three indicators showed the same variability and were again lowest in EP [1.7 (0.2, 9.6), 6.0 (0.6, 32.9), and 100.0 (95.7, 100.0) ml/kg/day, respectively, most Pcorrected < 0.001]. However, average HM-DD was lower only in EP than in LP [39.5 (19.4, 59.6) vs. 49.5 (27.2, 67.5) ml/kg/day, *P*_corrected_ < 0.001]. From days 1 to 28, average HM-DD in LP was 87.8 (60.2, 103.7) ml/kg/day, which was higher than PP 75.5 (47.8, 100.8) ml/kg/day and EP 52.6 (36.7, 87.5) ml/kg/day (both *P*_corrected_ < 0.001). For the first time in EP, average MOM-DD was significantly lower than in LP [43.1 (27.1, 80.0) vs 69.2 (44.3, 91.8) ml/kg/day, *P*_corrected_ = 0.010], although cumulative MOM-PCT was not statistically different. Also, at this stage, average DM-DD and cumulative HM-PCT in EP were significantly lower than in PP and LP. In conclusion, average DM-DD and cumulative HM-PCT were statistically different among the three hospitalization periods.

In addition to evaluating breastfeeding status using the above-mentioned quantitative indicators, the study also compared changes using common categorical evaluation indicators (except time to achieve full enteral feeding described in the following section, Table 5). Comparisons between the following variables were analyzed based on median values. In PP, all preterm infants ever received HM feeding within 3 days, while this decreased to 82.5% in EP and then recovered to 98.8% in LP (*P*_corrected_ < 0.05). Preterm infants in EP also had a lower percentage of HM-Ever within 7 days, at 95% (*P* < 0.05 overall, but *P*_corrected_ > 0.05 for EP vs LP). The rate of achieving cumulative HM-PCT ≥ 50% in EP was lowest within 14 days (EP, 91.8% vs PP, 99.4% or LP, 100.0%, *P*_corrected_ < 0.05). The rate of achieving HM-DD ≥ 50 ml/kg/day in EP was 53.1% within 28 days, smaller than in PP (72.3%) and LP (81.7%), *P*_corrected_ < 0.05. The rate of exclusive HM feeding in EP within 28 days was significantly lower than in PP and LP (EP, 42.9% vs PP, 81.7% or LP, 82.4%, *P*_corrected_ < 0.05). The rate of exclusive HM-DC was lower in EP than in the other two groups, but only significantly different from PP (EP, 78.7% vs. PP, 89.7% vs. LP, 88.9%, *P*_corrected_ < 0.05: between EP and PP). There was a difference in the time to achieve full enteral feeding between the three groups (*P* = 0.037); the median time in LP was 14 days, shorter than PP and EP of 15 days; however, this was not significantly different after Bonferroni correction.

## Discussion

During the COVID-19 global pandemic, the suspension of traditional bedside education models, concerns about the risk of vertical transmission of the virus, restricted family visits to the NICU, and limited milk delivery and donation have made it more challenging for preterm infants admitted to the NICU to receive adequate HM. Therefore, we implemented a new model of online lactation education to minimize interpersonal contact, reduce virus transmission, and break social distancing. We used the WeChat mini-program to educate parents about lactation during the pandemic and to provide timely and comprehensive lactation counseling to mothers. In a word, we managed the important mothers’ lactation period, wherein educators monitor the pumping frequency, milk output, and the key time points of lactation in the first 2 weeks postpartum online every day, and provide timely and reliable interventions to mothers with poor lactation status based on daily lactation data collected. This helps to maintain a healthy level of HM feeding during the NICU stay for preterm infants.

It was found that mothers using the model of online lactation education based on a WeChat mini-program were able to maintain a sufficient pumping frequency to achieve sustained growth in milk volume, with the majority of mothers achieving milk production ≥500 ml/d by day 14. In addition, there were no differences in maternal lactation indicators between EP and LP, further confirming that this education model can protect the normal lactation behavior in a lactation risk environment. Although HM feeding (both MOM and DM) of preterm infants was somewhat affected during the first 4 months of the pandemic (EP), this then recovered or even exceeded PP levels during the second 8 months (LP). Additionally, the record model based on the WeChat mini-program is more flexible, convenient, and economical than the traditional paper record method, and educators have dynamic access to the mother’s pumping details and enable online communication with less interpersonal contact. Similar breastfeeding enhancement programs exist for full-term mothers, and while they help mothers keep online pumping logs, they focus more on improving the usability of the application itself and have limited evaluation of their effectiveness (24–26). In contrast, our mini-program focuses on mothers of preterm infants. Regarding usability, the operation steps are very easy to follow, and there are also educators available to answer operational questions, as well as we are able to add or remove components of the app as needed. Moreover, our study describes in detail the effectiveness of the WeChat mini-program in terms of maternal lactation expression behavior and HM feeding status in preterm infants. In addition, the data of the mini-program can be linked to the online database, thus improving the efficiency of data integration. Altogether, data suggest that the WeChat mini-program is able to aid daily lactation education and management of high-risk groups (i.e., mothers of premature infants hospitalized in the NICU) to yield adequate milk output. Use of the mini-program in conjunction with optimized NICU management strategies may be able to ensure that milk supply is not compromised during a preterm NICU stay, thereby maintaining a healthy milk feeding level during this vulnerable period in the COVID-19 pandemic.

In EP and LP, mothers’ median daily pumping frequency was around eight times/d. Most evidence for pumping-dependent mothers generally recommends pumping 6-8 times a day (27). According to one study, mothers are recommended to pump at least eight times every day for at least 15 min, with a significant increase in daily and cumulative milk yield (28). In our study, the majority of mothers met this standard for daily pumping frequency except for the first day (probably because the pumping time on the first day was less than 24 h, and milk volume actually starts to increase substantially earlier; first-day time = 24 h – neonatal birth time). In addition, we found a statistical difference in mothers’ daily pumping frequency on day 1; however, mothers’ first day pumping time varied, so this difference was not very informative. On the second day, the daily pumping frequency in EP was slightly lower than in PP, most likely immature management strategies in EP and our early education process may have caused some impacts. By the third day, mothers generally started to feel breast fullness, which may have pushed them to maintain a higher pumping frequency and therefore did not show statistical differences on this day. Then, on days 4 and 5, the daily pumping frequency in PP was slightly lower than in EP, probably because most mothers were discharged during this time, which affected their milking schedule. In comparison to the traditional education model in PP, the use of mini-programs in LP could better urge mothers to pump milk in a timely manner, thus better solving this problem (this speculation is based on clinical experience and needs further confirmation). Finally, after several days of repeated education, mothers were able to maintain similar pumping frequency by days 6–14.

Mothers of preterm infants often encounter more difficulties in lactation than mothers of full-term infants and generally have insufficient lactation, thus requiring active manual intervention based on an understanding of the lactation process. The sequence of lactation stages includes initiation, CTV, and the maintenance of established lactation (9). The initiation and CTV are the most critical stages for the mother to establish lactation, which occurs within 14 days after delivery. In the first 3 days during the critical period of 14 days, the mammary gland transforms from preparing for lactation to secreting milk, which is the start of lactogenesis II and also the beginning of CTV. By 14 days, the daily milk output is at least 500 ml, meaning that lactation is established (i.e., CTV is achieved) (29, 30). The evaluation indicators of first milk expression time and first lactation time are related to lactation initiation, while OOL II-F, OOL II-Q, and percentage of achieved CTV are correlated with CTV.

There is currently some controversy about how early lactation should begin. According to “The Ten Steps to Successful Breastfeeding” (31), stimulation of lactation within 6 h increases mothers’ milk output postpartum. However, in recent years, a large study used a data-driven approach and identified the optimal lactation time to be within 8 h (32). The median first milk expression time of mothers in our study was 7–8 h. In many cases, the delay in early stimulation was due to a higher proportion of maternal cesarean delivery. Mothers need to be monitored for at least 6 h in the obstetric care unit, during which time lactation education cannot be performed early, but we plan to improve this by coordinating with obstetric nurses. We defined the time of first milk expression ≥1 ml as the onset of lactation, since it showed milk output emerging from none to ample amounts, and there was enough milk to perform breast milk oral care. Moreover, we performed oral care 4–5 times a day in the NICU, each of which used 0.2 ml of milk, meaning a total of 0.8–1 ml of a baby’s daily milk requirement. Breasts start to produce copious milk in lactogenesis stage II and mothers feel the breast milk “coming in,” accompanied by fullness, swelling, and a feeling of leakage. Feeling-related OOL II is the most commonly used indicator in research and clinical practice (33). However, since this method is subjective, a study proposed two consecutive milk production sessions ≥20 ml as a more practical definition (28). In the present study, the median time of OOL II-F or OOL II-Q in EP and LP was never >72 h (delayed lactogenesis II onset), indicating a better milk increase.

It suggested that milk output of at least 500 ml/day at 14 days can meet the exclusive MOM supply of preterm infants during hospitalization and is a strong predictor of continued MOM provision at NICU discharge. In addition, it decreases unexpected risks in lactation, so this intuitive indicator is used as a common criterion for evaluating short-term lactation success (29, 34, 35). During PP, EP, and LP, milk volume maintained a steady increase from days 1 to 14, achieving a median daily volume of at least 500 ml by day 14, but daily volumes were not significantly different between the three groups. The percentage of CTV achieved increased from 56.7% in PP to 60.7% in LP (although *P*_corrected_ > 0.05), much higher than the 39.5% reported previously (34). However, the population in that study included very low birth weight infants (VLBWI) and the mean gestational age was smaller. The percentage of CTV in another study was slightly higher than ours; however, although it included a similar population, highly efficient hospital-grade breast pumps were used (10).

Most of the existing breastfeeding indicators for preterm infants are based on categorical variables and are usually presented as the percentage to which a certain indicator has been achieved. For example, the percentage of those who had ever received MOM during hospitalization or the percentage of those who still received DM or MOM feeding at discharge is evaluated, among which the latter is widely used (36–38). In the present study, we compared breastfeeding in three groups using categorical evaluation indicators that are frequently used in the field. In this study, the rate of HM-Ever within 3 and 7 days both decreased in EP; exclusive HM feeding within 28 days and exclusive HM-DC also declined in EP, and the former decreased by nearly a half compared to PP and LP. However, the indicators of MOM feeding related to the above four parameters were not significantly different. This may be related to the reduction in milk donation due to the strict measures to control COVID-19 during the milk donation process. This problem has also arisen in other countries. One study mentioned that some hospitals have reduced orders for donated human milk, mainly due to financial and trust issues, and the pandemic also severely impacted how milk banks function (39). Another study also mentioned the impact of the pandemic on global breast milk banks, with a decrease in milk donations in some parts of India (40). In our study, the rate of exclusive MOM-DC was lowest in EP at 77.0%, but higher than the 35.2–46.0% mentioned by Ward et al. (41) for VLBWI. It was also similar to the 77.4% of the continuous improvement period reported in a quality improvement study (37). The rate of exclusive HM-DC reached 78.7–89.7% in our study, higher than the percentage (58.3%) established in a previous study (42). In addition, the rate of partial HM-DC of VLBWI reported by 14 NICUs in New Jersey in 2016 was 55% (43), which was lower than our exclusive HM-DC rate. Meanwhile, the HM-Ever rate within 7 days mentioned in that study was 91%, slightly lower than our rate of 95–100%. Of the 32 VLBWI, 21 were exclusively on MOM feeding for the first 28 days in a quality improvement program of a NICU (44). Nevertheless, the present study has shown that the rate of infants who were exclusively MOM feeding within 28 days was quite low.

However, categorical evaluations of breastfeeding, such as feeding types at discharge (exclusive HM, exclusive MOM, exclusive FM, or mixed), included only those babies who had accepted a certain type of feeding, and then the rate was calculated, which ignores the time and volume of feeding. Nevertheless, studies have shown that the use of a certain amount of HM for a specific period is strongly associated with a reduced risk of morbidities in VLBWI. This dose-dependent effect of breast milk underscores the need for a quantitative evaluation of VLBWI breastfeeding during NICU hospitalization (23). Therefore, we used the average HM-DD and cumulative HM-PCT (MOM and DM were included) values of quantitative evaluation indicators to evaluate breastfeeding. We were surprised to find that the average HM-DD within 28 days was highest in LP after using the mini-program to manage mothers’ lactation. Median average MOM-DD and cumulative MOM-PCT between 1 and 28 days were lowest in EP at 43.1 ml/kg/day and 75.7%, respectively, while in LP they were 69.2 ml/kg/day and 89.8%, respectively. These two values were 45.7 ml/kg/day and 98% in the study of Bigger et al. (23) (DM was not used in this study, so HM = MOM). In another study, the median average MOM-DD within 28 days in VLBWI was 79–117 ml/kg/day (45). However, in that study, the weight-adjusted daily dose was divided by birth weight and not daily weight, which may lead to higher values. In addition, when adding the extra DM, median average HM-DD and cumulative HM-PCT within 28 days in LP were 87.8 ml/kg/day and 100%, respectively, which suggested good HM feeding rates in the present study.

Some studies also use special categorical indicators derived from the above-mentioned quantitative indicators. It is suggested that cumulative HM-PCT ≥ 50% within 14 days reduces the incidence of NEC in VLBWI by six-fold (46), while cumulative MOM-PCT ≥ 50% within 14 days was predictive of continued breastfeeding at 3 months corrected gestational age in preterm mothers (47). Furthermore, HM-DD ≥ 50 ml/kg/day within 28 days reduces the incidence of sepsis in VLBWI while reducing hospitalization costs (48). In the present study, the rates of cumulative HM-PCT ≥ 50% and HM-DD ≥ 50 ml/kg/d were both lowest in EP, although with no significant differences in PP and LP. Thus, we conclude that although breastfeeding in preterm infants is influenced by a variety of factors and there were some differences in the populations between the above studies, breastfeeding returned to a relatively healthy level in LP.

In conclusion, this study applied two methods, quantitative indicators and categorical indicators, to assess changes in breastfeeding before the pandemic and during the early and late pandemic when a WeChat mini-program was used to manage lactation. Many indicators decreased in EP, but in LP, with the implementation of normalized management in conjunction with the mini-program, they recovered or even exceeded the level of the PP period. Achieving full enteral feeding early reduces the need for central venous catheters, which further reduces the risk of infection and shortens the hospital stay (49). The median time to achieve full enteral feeding in this study was 14 days in LP, which was 1 day shorter than in PP and EP (although the difference between groups was not significant after correction). The average time to achieve full enteral feeding in a previous study was 20–24 days (50), and in another study, the time in infants < 32 weeks decreased from 12.8 days before quality improvement to 7.7 days after quality improvement (51), suggesting that we still have room for improvement.

We used a WeChat mini-program, which has a huge user base in China, for our online expression education. This allowed us to break social distancing to communicate during COVID-19, greatly reducing direct contact and the spread of the virus. Mothers are often weak after delivery, and the education effect is usually poor. Our first mission was primarily for fathers in the “education room,” which is beneficial to increase mothers’ rest time and facilitate lactation. We created a motivational mechanism to acquire photos of babies that greatly reduced maternal anxiety and enhanced maternal motivation to lactate, although this effect was not carefully studied in this project. With the WeChat mini-program, mothers can provide timely feedback on their lactation challenges through the online communication function, and they can also keep track of daily pumping frequency and milk volume. This lactation education model applies the “ecological momentary assessment” method, which allows for subtle measurements of human behaviors and real-time interventions while minimizing recall bias (25). The first face-to-face mission is mainly targeted at fathers, whose psychological and practical support influences the initiation and duration of breastfeeding for mothers, and improves the rate of exclusive breastfeeding at 4 and 6 months (52). Moreover, educators are able to manage multiple mothers in early lactation at the same time with the help of only one cell phone, which saves time and increases their work efficiency.

There are some limitations in this study. Since it was not possible to identify the exact timing of the effect of the pandemic on breastfeeding in our hospital, we had to approximate a larger time range to include the period most affected by the pandemic. This has little impact on the study, since its main purpose was to investigate breastfeeding in preterm infants with the application of a WeChat mini-program. The Bonferroni correction used for pairwise comparisons may lead to an increased probability of Type II error (real differences are considered no differences), but the multiple comparison numbers were small in this paper (only three times), so the corrected results were still relatively reliable. Notably, both an increase in maternal milk volume and changes in lactation indicators may benefit from the WeChat mini-program, but it may also be caused by the mothers’ behavior returning to normal in EP, and this can be further investigated in the subsequent studies. Also, different educators might influence the results, but our educational process was standardized and the educators had been uniformly trained, so this factor had little influence. Furthermore, the study only assessed short-term breastfeeding, and given that the World Health Organization recommends exclusive breastfeeding until at least 6 months, later breastfeeding follow-up is also necessary. In addition, the lactation data were self-administered and may be subject to some bias, but similar data collection methods were applied in similar studies. What is more, this study was conducted in a tertiary hospital in the more economically developed eastern region, so the general applicability is not strong, but other colleagues are more than welcome to use our mini-program for breastfeeding promotion or similar studies.

## Conclusion

Breastfeeding in NICUs for preterm infants is challenging under global health emergencies, such as the COVID-19 global pandemic. Although breastfeeding was somewhat affected in the early stage of the pandemic, the online lactation education model based on a WeChat mini-program could break social distancing and convey health education knowledge while reducing interpersonal contact. Moreover, human breastfeeding can be restored to pre-pandemic levels, or even increased beyond these. In future research, the WeChat mini-program can be used not only for long-term breastfeeding follow-up, but also for prenatal breastfeeding education for mothers, and even for research on mothers’ attitudes and confidence in breastfeeding.

## Data Availability Statement

The raw data supporting the conclusions of this article will be made available by the authors, without undue reservation.

## Author Contributions

CJ, SH, and ZY designed the study. CJ and XuC collected the data. CJ drafted the manuscript. SH and ZY revised the manuscript. All authors worked together to verify the validity of all data and provide data analysis and read and approved the final version of the manuscript.

## Conflict of Interest

The authors declare that the research was conducted in the absence of any commercial or financial relationships that could be construed as a potential conflict of interest.

## Publisher’s Note

All claims expressed in this article are solely those of the authors and do not necessarily represent those of their affiliated organizations, or those of the publisher, the editors and the reviewers. Any product that may be evaluated in this article, or claim that may be made by its manufacturer, is not guaranteed or endorsed by the publisher.

## Footnotes

1
http://www.ningbx.com
